# Patterns and clinical significance of cervical lymph node metastasis in papillary thyroid cancer patients with Delphian lymph node metastasis

**DOI:** 10.18632/oncotarget.19047

**Published:** 2017-07-06

**Authors:** Guibin Zheng, Hua Zhang, Shaolong Hao, Chengxin Liu, Jie Xu, Jinyao Ning, Guochang Wu, Lixin Jiang, Guojun Li, Haitao Zheng, Xicheng Song

**Affiliations:** ^1^ Department of Thyroid Surgery, The Affiliated Yantai Yuhuangding Hospital of Qingdao University, Yantai, Shandong Province, 264000, China; ^2^ Department of Otolaryngology-Head and Neck Surgery, The Affiliated Yantai Yuhuangding Hospital of Qingdao University, Yantai, Shandong Province, 264000, China; ^3^ Departments of Head and Neck Surgery, The University of Texas MD Anderson Cancer Center, Houston, TX 77030, U.S.A.; ^4^ Department of Epidemiology, The University of Texas MD Anderson Cancer Center, Houston, TX 77030, U.S.A.

**Keywords:** Delphian lymph node, papillary thyroid cancer, central neck node metastases, lateral neck node metastases

## Abstract

Although the roles of Delphian lymph node (DLN) metastasis in papillary thyroid cancer (PTC) have been previously reported, there are still limited data on correlations of clinicopathologic factors with DLN metastasis and unique patterns of cervical node subsite metastasis in PTC patients with DLN metastasis. We retrospectively reviewed medical records of 320 patients with a diagnosis of PTC who underwent primary surgery. Clinicopathologic features and DLN metastasis patterns were analyzed for predicting extensive cervical lymph node metastasis. Both univariate and multivariate Cox regression analyses were used to identify independent factors for cervical lymph node metastasis. DLN metastasis was significantly associated with multifocality, tumor size > 1 cm, extrathyroid extension, BRAF^V600E^ mutation, central neck node metastasis (CNNM), and lateral neck nodes metastases. Patients with DLN metastasis had more lymph node metastases in the central compartment. CNNM number and tumor size > 1 cm were independent risk factors for DLN metastasis. DLN metastasis was highly predictive of lateral lymph node metastasis with moderate sensitivity and high specificity. DLN metastasis is associated with several poor prognostic factors, including extensive cervical lymph node metastasis, and can serve as a predictor of advanced PTC. The presence of DLN metastasis should prompt surgeons to perform an aggressive surgery approach.

## INTRODUCTION

Central neck nodes (CNNs; also known as level VI nodes), which comprise the Delphian (prelaryngeal), pretreacheal, and paratracheal node groups, are the most common harbors of nodal metastasis in patients with papillary thyroid cancer (PTC). The percentage of PTC patients with central neck node metastasis (CNNM) at primary diagnosis ranges from 20% to 53.7% [1–6]. Although some authors have reported that CNNM has no major impact on the survival rate of low-risk PTC patients [3, 7], emerging evidence suggests that CNNM has an adverse effect on the locoregional recurrence and survival rates of intermediate- and high-risk patients [2, 4, 8]. Hence, prophylactic central neck dissection (CND) of the affected side should be performed following thyroidectomy in intermediate- and high-risk PTC patients, as it may improve survival by reducing locoregional recurrence and facilitating accurate disease staging [9–11]. However, given the high incidence of laryngeal nerve palsy and hypoparathyroidism following bilateral CND, whether these patients should also have contralateral CND is still a debated issue.

Currently, lateral node metastasis is received more attention. And lateral neck dissection (LND) is recommended for therapeutic purposes. In fact, PTC patients have been reported to have a high frequency of occult lateral node metastasis [12, 13]. However, the indications for prophylactic LND in PTC patients are controversial and remain unclear, as data for finding an optimal balance between its complications and benefits are limited.

**Figure 1:**
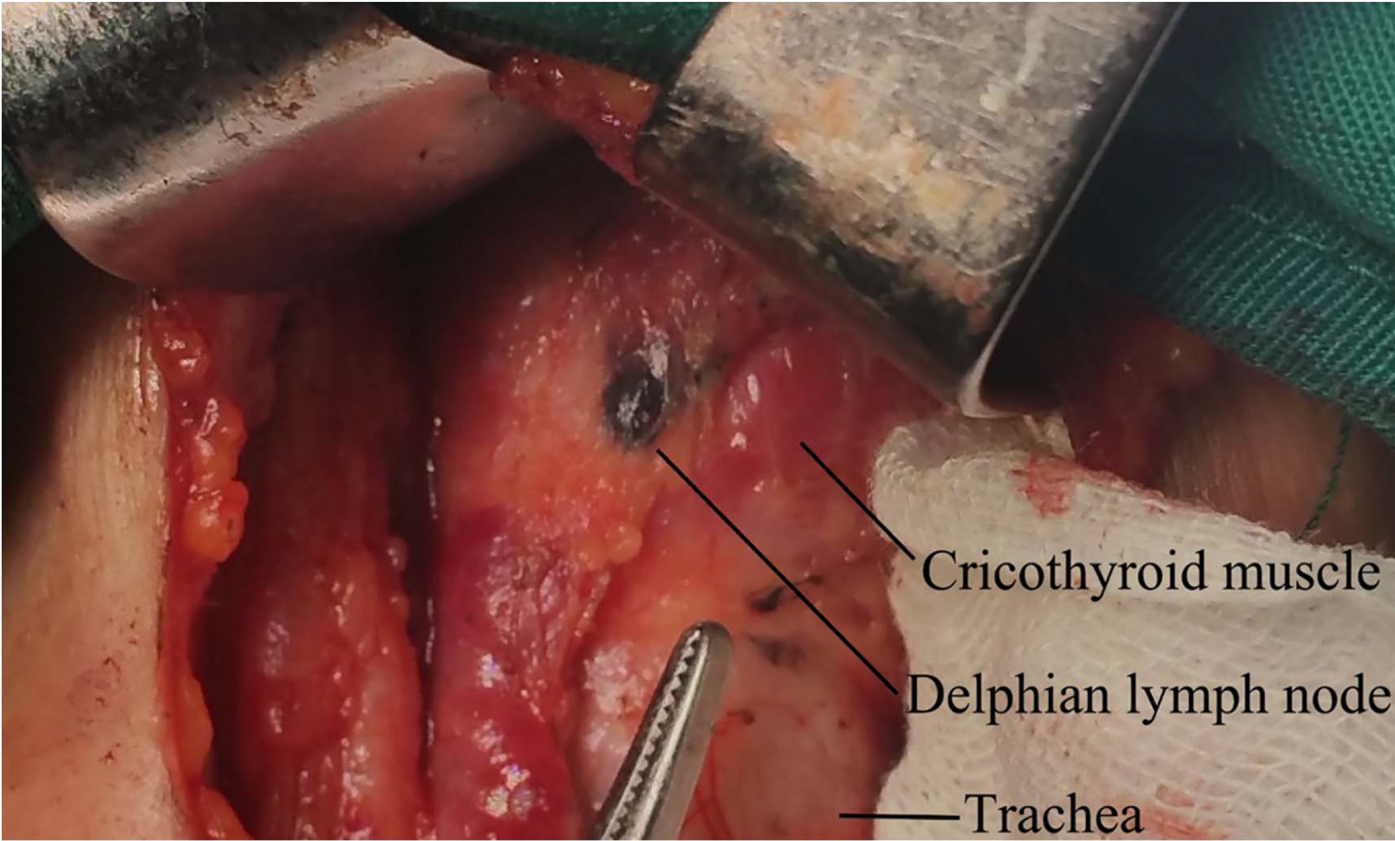
The Delphian lymph node is darkened by nanocarbon

Disease involvement of the Delphian lymph node (DLN), which is located anterior to the cricothyroid membrane, between the cricothyroid muscles (Figure 1), is a poor prognostic predictor in many malignant neck cancers [14, 15]. Thus, DLN status has important implications in planning appropriate surgical treatment and determining outcome. To our knowledge, only 8 studies have investigated the role of DLN in thyroid cancer management [16–23]. With the exception of extensive cervical lymph node metastasis, the clinicopathologic factors these studies report to be associated with DLN metastasis are inconsistent. However, owing to the limited data on DLN metastasis in PTC, its relationship with other nodal metastasis in the central and lateral compartments and its clinical significance are still debated. Hence, the aim of the present study was to assess the risk factors for DLN metastasis and investigate the pattern of cervical LNM to provide information for lymph node dissection in PTC patients with DLN metastasis.

## RESULTS

Of the 320 patients included in the study, 157 (49.1%) had CNNM, and 4 (1.3%) had DLN metastasis without metastasis to other central compartments. The DLN was detected in 206 patients (64.4%), among whom, 42 patients (20.4%) had confirmed DLN metastasis (Table 1). The percentage of DLN metastasis in patients with PTC was 13.1%. Thyroid follicular tissue was detected in 71 patients (22.2%) and the remaining showed with fibro-adipose tissue. One patient had PTC in the prelaryngeal region without any tumor foci in the thyroid gland and was excluded from the analysis.

**Table 1:** Rates of Delphian lymph node (DLN) detection and metastasis

Variables	N/total (%)*
DLN detection	206/320 (64.4)
DLN metastasis	42/206 (20.4)
Central neck node metastasis	157/320 (49.1)
Mean no. of DLNs (range)	1.7 (1–6)
Mean no. of DLN metastases (range)	1.4 (1–4)

*Unless otherwise indicated.

The univariate analysis revealed that, compared with patients without DLN metastasis, those with DLN metastasis had significantly higher rates of multifocality (47.6% vs. 28.0%, *P* = 0.015), tumor size > 1 cm (71.4% vs. 31.1%, *P* < 0.001), ETE (59.5% vs. 37.2%, *P* = 0.009), BRAF^V600E^ mutation (64.3% vs. 47.6%, *P* = 0.026), paratracheal LNM (81.0% vs. 31.3%, *P* < 0.001), pretracheal LNM (64.3% vs. 23.2%, *P* < 0.001), and CNNM (90.5% vs. 41.5%, *P* < 0.001) and significantly more CNNMs (4.3 ± 3.0 vs. 2.3 ± 1.5, *P* < 0.001). (Table 2).

**Table 2:** Comparison of clinicopathological characteristics between DLN-positive/negative patients

Variable	No. of DLN-positive patients (%), *n* = 42	No. of DLN-negative patients (%), *n* = 164	*P* value
Age ≥ 45 years	17 (40.5)	86 (52.4)	0.167
Sex			
Female	29 (69.0)	130 (79.3)	0.429
Male	13 (31.0)	34 (20.7)	
Multifocality	20 (47.6)	46 (28.0)	0.015
Tumor size >1 cm	30 (71.4)	51 (31.1)	0.001
ETE	25 (59.5)	61 (37.2)	0.009
Thyroiditis	15 (35.7)	58 (35.4)	0.966
Tumor location			
Isthmus/upper third	18 (42.9)	76 (46.3)	0.923
Middle/lower third	24 (57.1)	88 (53.7)	
BRAF^V600E^ gene			
Mutant	27 (64.3)	78 (47.6)	0.026
Wild type	7 (16.7)	18 (11.0)	
Undetected	8 (19.0)	68 (41.5)	
Paratracheal LNM	34 (81.0)	51 (31.1)	0.001
Mean no. of paratracheal LNM ± SD	3.2 ± 2.1	1.8 ± 1.1	0.001
Pretracheal LNM	27 (64.3)	38 (23.2)	0.001
Mean no. of pretracheal LNM ± SD	2.0 ± 1.6	1.6 ± 0.8	0.142
CNNM	38 (90.5)	68 (41.5)	0.001
Mean no. of CNNM ± SD	4.3 ± 3.0	2.3 ± 1.5	0.001

Note: Data are no. of patients (%) unless otherwise indicated.

DLN: Delphian lymph node; ETE: extrathyroid extension; LNM: lymph node metastases; CNNM: central neck node metastases.

The multivariate logistic analysis revealed that, compared with patients without DLN metastasis, those with DLN metastasis had significantly higher rates of tumor size >1 cm (71.4% vs. 31.1%, *P* = 0.008) and significantly more paratracheal LNM (3.2 ± 2.1 vs. 1.8 ± 1.1, *P* < 0.001) and CNNM (4.3 ± 3.0 vs. 2.3 ± 1.5, *P* < 0.001). (Table 3).

**Table 3:** Multivariate logistic regression analysis of Delphian lymph node metastasis

Covariates	Adjusted odds ratios	95% confidence interval	*P* value
Multifocality	1.1	0.4–2.6	0.908
Tumor size > 1 cm	3.5	1.4–8.7	0.008
ETE	1.6	0.6–3.9	0.326
BRAF^V600E^ mutation	0.7	0.2–2.4	0.556
No. of paratracheal LNM	2.0	1.4–2.8	0.001
No. of pretracheal LNM	1.4	0.9–2.1	0.217
No. of CNNM	1.7	1.4–2.1	0.001

ETE: extrathyroid extension; LNM: lymph node metastases; CNNM: central neck node metastases.

Thirty patients underwent modified neck dissection of ≥ 1 lateral node sublevel. Skip metastases (positive LNN but negative CNN) were detected in 4 of 27 patients (15%) without DLN metastasis and 1 of 18 patients (6%) with DLN metastasis. LNNM was found in 14 of 14 patients (100%) with DLN metastasis and 4 of 7 patients (57%) without DLN metastasis, a significant difference (*P* = 0.029). Patients with DLN metastasis and LNNM had more CNNM than patients without DLN metastasis but with LNNM did. (Table 4).

**Table 4:** Clinicopathological characteristics of patients with lateral neck lymph node metastasis (LNM)

Variables	No. of DLN-positive patients (%), *n* = 14	No. of DLN-negative patients (%), *n* = 4	*P* value
Mean no. of LNN removed ± SD	19.8 ± 12.1	18.3 ± 2.2	0. 121
Mean no. of LNNM ± SD	4.1 ± 1.6	4.3 ± 2.6	0.227
BRAF^V600E^ gene			
Mutant	10 (71)	3 (75)	0.887
Wild type	4 (29)	1 (25)	
ETE	11 (80)	4 (100)	0.196
Mean tumor size ± SD, cm	2.0 ± 0.9	1.4 ± 0.5	0.208
Multifocality	8 (57)	4 (100)	0.245
Mean no. of CLN removed ± SD	7.7 ± 4.3	9.3 ± 4.6	0.540
Mean no. of central LNM ± SD	6.0 ± 3.1	2.3 ± 2.6	0.044

Note: Data are no. of patients (%) unless otherwise indicated.

DLN: Delphian lymph node; LNN: lateral neck node; LNNM: lateral neck node metastasis; ETE: extrathyroid extension; CLN: central lymph node; LNM: lymph node metastasis.

Of the 14 patients with DLN metastasis who underwent LND, 11 (79%) had multiple levels with nodal metastasis. Level III was the most frequently involved (86%), followed by level IV (71%), and level II (43%). Only 2 of these patients (14%) had metastases in level V nodes. (Table 5).

**Table 5:** Metastasis pattern in 14 patients with Delphian lymph node metastasis and lateral neck metastasis

Lateral neck sublevel	No. of patients with positive nodes (%)
Level II, upper jugular	6 (42.8)
Level III, middle jugular	12 (85.7)
Level IV, lower jugular	10 (71.4)
Level V, posterior triangle	2 (14.3)
Multiple levels with nodal metastasis	11 (78.6)

Compared with patients without DLN metastasis, patients with DLN metastasis had more CNNMs (4.3 ± 3.0 vs. 2.3 ± 1.5, *P* < 0.001) (Table 2) and were 14 times more likely to have further CNNM. (Table 6).

**Table 6:** Ability of Delphian lymph node metastasis to predict paratracheal, pretracheal, central, and lateral lymph node metastasis (LNM)

LNM types	Sensitivity (%)	Specificity (%)	PPV (%)	NPV (%)	LR+	LR-
Paratracheal	37.0	93.4	81.0	66.1	5.6	0.7
Pretracheal	41.5	89.4	64.3	76.8	3.9	0.7
Central	55.8	96	90.5	58.5	14.0	0.2
Lateral	77.8	100	100	21.4	77.8*	0.8*

LNM: lymph node metastasis; PPV: Positive predictive value; NPV: Negative predictive value; LR+: positive likelihood radio; LR-: negative likelihood radio.

*Specificity assigned as a value of 0.99 to permit calculation of LR+ and LR-.

The incidence of ipsilateral paratracheal LNM and contralateral paratracheal LNM among patients with DLN metastasis (100% and 50% separately) were higher than that among patients without DLN metastasis (45.5% and 27.3% separately).The difference of ipsilateral paratracheal LNM was statistically significant (*P* = 0.043), while the contralateral paratracheal LNM was not (*P* =0.6). (Table 7).

**Table 7:** Ipsilateral or contralateral paratracheal lymph node metastasis (LNM) in DLN-positive/negative patients

LNM types	No. of DLN-positive patients (%), *n* = 6	No. of DLN-negative patients (%), *n* = 11	*P* value
Ipsilateral paratracheal	6 (100)	5 (46)	0.043
Contralateral paratracheal	3 (50)	3 (27)	0.600

LNM: lymph node metastasis; DLN: Delphian lymph node.

Bilateral PTC was observed in 14(60.9%) of DLN-positive patients and 25(34.7%) of DLN-negative patients, and the difference was significant (*P* = 0.026). Contralateral tumor foci without suspicion preoperatively by US were identified in 9(39.1%) of DLN-positive patients, and 12(16.7%) of DLN-negative patients, which had significant difference (*P* = 0.024). The tumor sizes of two groups were too small and no significant difference was found. (Table 8).

**Table 8:** Preoperative status of contralateral nodules on US in DLN-positive/-negative patients with total thyroidectomy

Variables	No. of DLN-positive patients (%), *n* = 23	No. of DLN-negative patients (%), *n* = 72	*P* value
Bilateral PTC	14 (60.9)	25 (34.7)	0.026
Preoperative status of contralateral nodules on US			
Unsuspected	9 (39.1)	12 (16.7)	0.024
Tumor size(cm)*	0.40 ± 0.17	0.33 ± 0.20	0.768

US: ultrasonography; DLN: Delphian lymph node

*The size of the contralateral PTC foci that was unsuspected on preoperative US.

**Figure 2:**
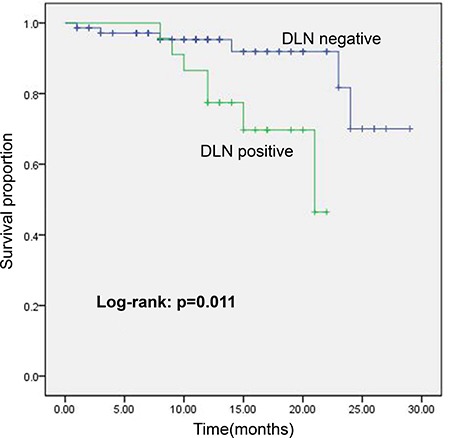
Kaplan Meier plot showing cumulative survival of Tg ≥ 1 ng/ml in DLN-positive/-negative patients with total thyroidectomy in the follow-up period

Serum thyroglobulin (Tg) of the patients with total thyroidectomy was reviewed after surgery for recurrence monitoring. The median of follow-up was 14 months (range: 8–22 months) and 11 months (range: 1–26 months) for DLN-positive patients and DLN-negative patients, respectively. Unstimulated Tg ≥ 1ng/ml was chosen to evaluate the likelihood of having persistent or recurrent disease in response to an initial surgery, since an unstimulated postoperative Tg < 1 ng/ml was associated with favor outcomes with recurrence rates < 1% [24]. Kaplan Meier analysis showed that DLN-positive patients had significantly a higher rate of unstimulated Tg ≥ 1 ng/ml than DLN-negative patients (30.4% vs. 6.9%, *P* = 0.011) as shown in Figure 2. Moreover, disease recurrence was detected on follow-up by US and confirmed by FNA or RAI scaning in 3 DLN-positive patients with unstimulated Tg > 20 ng/ml. The unstimulated Tg levels in other patients with unstimulated Tg≥1ng/ml were all less than 8 ng/ml; and all these patients were disease recurrence-free after treatment. There were no cases for distant metastasis.

## DISCUSSION

The DLN receives lymphatic drainage from the larynx and thyroid, then drains to the lateral neck nodes through the superior thyroid artery. The median size of DLN metastasis deposits is 0.3 cm [18]; thus, DLN status is difficult to evaluate preoperatively with ultrasonography, computed tomography, or magnetic resonance imaging [15, 22]. Therefore, DLN status must be assessed by frozen section biopsy intraoperatively to provide information about whether further CND is warranted. DLN excision can be easily performed before thyroidectomy and does not prolong the time of operation or hamper other nodal dissection in the central compartment. It has been reported that DLN metastasis can be used to predict extensive LNM, recurrence, and shorter survival in patients with laryngeal, glottis, or hypopharyngeal cancer [14, 15]. Although the DLN is the first lymph node encountered and could be easily resected during thyroidectomy, whether the DLN can have the same role in thyroid cancer patients is still controversial because of insufficient data.

To the best of our knowledge, only 8 previous studies have investigated the significance of DLN status in thyroid cancer patients [16–23]. In these studies, a DLN was detected in 23.0%–74.6% of patients with thyroid cancer, and of those DLNs, 8.2%–24.8% had metastatic disease. In our study of PTC patients, the DLN detection and metastasis rates were 64.4% and 20.4%, respectively, which are similar to those reported by Kim et al. (74.6% and 17.2%, respectively) [19]. Our study had a high rate of DLN detection because it enrolled only PTC patients whose initial treatment was surgery.

All 8 previous studies indicated that DLN metastasis is associated with concomitant cervical LNM, but the clinicopathological factors they reported to be associated with DLN metastasis were not consistent. These factors included multifocality [19, 21], autoimmune thyroiditis [25], tumor location [20, 22], lymphovascular invasion [19, 22], ETE [18–20, 22, 23], larger tumor size [16, 19–23], and cervical LNM [16–23]. Four of the eight previous studies included multivariable analyses, which identified CNNM [19, 21, 22], lymphovascular invasion [19], and tumor location in the isthmus or upper third of the thyroid [22], larger tumor size [21], and ETE [23] as independent risk factors for DLN metastasis. In addition, we investigated for the first time the relationship between DLN metastasis and BRAF^V600E^ mutation, a risk factor for neck lymph node metastasis and poor outcome [26]. Our results showed that DLN metastasis is correlated with BRAF^V600E^ mutation (correlation coefficient = 0.180, *P* < 0.01), although the odds ratio for BRAF^V600E^ mutation in the multivariable analysis was 0.7, which may have been due to the great differences in the sample sizes and BRAF^V600E^ mutation detection rates of the groups with or without DLN metastasis. Moreover, compared with patients without DLN metastasis, patients with DLN metastasis had more CNNMs (*P* < 0.001) and were 14 times more likely to have further CNNM. Taken together, these findings indicate that PTC metastasis to the DLN is associated with several poor prognostic factors, including ETE, tumor size >1 cm, BRAF^V600E^ mutation, and CNNM. According to management guidelines for thyroid cancer, these factors greatly contribute to an intermediate or high risk of disease recurrence [27].

The extent of CND for PTC is still controversial and the exploration of the relationship between DLN metastasis and other CNN subsites would provide important information for surgeon. CNNs are the first nodes to harbor metastatic PTC, and the number of CNNMs is correlated with high recurrence risk and lower overall survival [4, 28]. In PTC patients, the paratracheal and pretracheal lymph nodes are the most common subsites in the central compartment to harbor metastasis [6, 17, 29]. Our data showed that DLN-positive patients had significantly higher rates of paratracheal LNM (81.0% vs 31.1%, *P* < 0.001) and were 5.6 times more likely to harbor paratracheal LNM than DLN-negative patients. Previous studies showed that, among patients with DLN metastasis, 42.9%–84.6% have ipsilateral paratracheal LNM, whereas 1.7%–9.8% have contralateral paratracheal LNM [10, 30, 31]. The negative predictive value of not having contralateral paratracheal LNM in the absence of DLN metastasis is 90.3% [19]. These results suggest that CND on the affected side (including DLN, ipsilateral paratracheal lymph nodes, and pretrecheal lymph nodes) should be routinely performed in DLN-positive patients, while ipsilateral CND is sufficient for DLN-negative patients.

Contralateral CND (including contralateral paratracheal lymph nodes), as part of bilateral CND, can be performed on the premise of total thyroidectomy. In our study, bilateral PTC was observed to have a significant difference between DLN-positive patients and DLN-negative patients. Contralateral tumor foci without suspicion preoperatively by US were significantly higher than that of DLN-negative patients. The tumor sizes of two groups were too small and no significant difference was found. Thus, we recommended that total thyroidectomy should be performed in DLN-positive patients with nodules in contralateral lobe in order to remove all the tumor foci unsuspected preoperatively by US. It was reported that contralateral paratracheal LNM was observed in 13.3%-21.0% of patients with PTC and associated with ipsilateral CNNM and DLN metastasis (P = 0.028) [32, 33]. Kim et al. reported that the incidence of contralateral paratracheal LNM among DLN-positive patients (41.9%) is significantly higher than that among DLN-negative patients (9.6%; *P* < 0.001) regardless of tumor size. The same results was observed in PTMC between the two groups (41.7% vs 6.6%, *P* < 0.001) [19]. Our data also showed the same tendency (50% vs 27%), although it had not a significant difference, likely because of the small number of patients with bilateral paratracheal lymph node dissection. It was suggested that it’s very likely to suffer from LNM in contralateral paratracheal region when PTC metastasize to DLN. Moreover, the recurrence rate of contralateral paratracheal lymph node with a mean of follow-up of 12.1 years was 1.8% in patients who received total thyroidectomy and ipsilateral CND at initial surgery [34]. Since bilateral CND, which had decreased CNN recurrence, can be performed safely by experienced surgeons at high-volume centers [35, 36], contralateral CND should be considered in DLN-positive patients with intermediate or high risk of recurrence. If contralateral paratracheal lymph node dissection cannot be performed, such patients’ follow-up care should include careful surveillance of the contralateral CNN.

Currently, lateral neck dissection is performed for therapeutic purposes only. Lateral neck dissection should include the removal of at least the level IIa, IIb, III, and IV nodes, as these are the most common locations of metastatic disease, which is found in level III nodes in 57.0%–74.8% of patients, level IV nodes in 51.0%–75.9% of patients, and level II nodes in 52.0%–72.2% of patients [31, 37, 38]. Moreover, 46.1%–80.7% of patients have lateral nodal disease at multiple levels [31, 38]. Previous studies have shown that DLN metastasis is a risk factor for lateral nodal metastasis [16–19]. In the present study, of the 42 patients with DLN metastasis, 14 (33%) had preoperative evidence of lateral node involvement, and metastatic disease to lateral nodes at multiple levels was observed in 11 (78.6%) DLN-positive patients with LNNM. These results indicate that DLN metastasis is associated with extensive nodal metastasis in the lateral neck compartment and that patients with DLN and lateral node metastasis should undergo careful LND. The relationship between DLN and occult LNNM in patients with PTC has not been discussed yet. There are no clear indications for performing LND in patients with no preoperative evidence of LNNM. Fraser et al. reported that 41 of 137 PTC patients (30%) had occult disease in just level III [13]. Moreover, Mazzaschi et al. found that 32 of 40 patients (80%) with differentiated thyroid cancer who did not have preoperative evidence of cervical LNM had ipsilateral CNNM and LNNM simultaneously [29]. These studies highlight the great discrepancy between the high frequency of pathological LNNM and low rate of clinical lateral nodal involvement. Other studies have reported that DLN metastasis is highly predictive of LNNM [16–19], which is consistent with our results. Several of those studies reported that patients with DLN metastasis are 3.5–8.8 times more likely than patients without DLN metastasis to have LNNM [16–18]. In addition, because patients with DLN metastasis have extensive CNNM, it is reasonable to assume that a number of patients with DLN metastasis have occult LNNM. Hence, prophylactic LND may benefit patients with DLN metastasis by reducing the risk of regional recurrence and improving survival [39, 40]. Given the significant morbidity of LND, patients with DLN metastasis but no evidence of lateral nodal involvement may benefit from sentinel node biopsy, which has demonstrated promising results [41, 42], for evidence of occult metastasis and justification of LND.

Several limitations of our study should be considered. First, the relationship between DLN and lymphovascular invasion was not evaluated because this information cannot obtained from the pathology reports of the patients, which lead to a loss of reference in the analyses of the relationship between DLN metastasis and other nodal metastasis, although only 2 studies reported an association between DLN metastasis and lymphovascular invasion [19, 22]. Second, the sample size of cases underwent bilateral CND and LND is small, which may cause a bias in our results. As PTC is an indolent tumor and short-time follow-up could not get enough information, further studies with larger patient population and longer follow-up period are warrant to justify the clinical significance of DLN metastasis.

In conclusion, our findings demonstrate that once PTC has metastasized to the DLN, the disease has become aggressive, with several poor prognostic factors that predict an intermediate or even high risk of recurrence. Because patients with DLN metastasis have extensive CNNM and a high risk of LNNM, such patients’ DLNs should be assessed intraoperatively with frozen section biopsy which may help determine an aggressive surgery approach.

## MATERIALS AND METHODS

### Study patients

We retrospectively reviewed the medical records of 320 patients with a final diagnosis of PTC at primary surgery performed between August 2014 and May 2016 in the Thyroid Surgery Department and Otorhinolaryngology-Head & Neck Surgery Department at Qingdao University Affiliated Yantai Yuhuangding Hospital, China.

The study protocol was approved by the Committee of Ethics in Research of Yuhuangding Hospital of Qingdao University. All of the following procedures were in accordance with the ethical standards of the responsible committees for human experimentation (institutional and national) and with the Helsinki Declaration of 1975, as revised in 2008.

Tissues with DLNs were excised in all 320 cases, then were labeled as DLNs and examined by separate histopathological examinations. DLNs were detected in 206 patients, and DLN metastasis was confirmed in 42 of these patients. We investigated the relationship between DLN metastasis and gender, age, tumor size, multifocality, thyroiditis, BRAF^V600E^mutation, extrathyroid extension (ETE), tumor location, CNNM, and lateral neck node metastasis (LNNM). The central neck lymph node classification was assigned, except DLN, to investigate the relationship with further metastasis in other central subsites.

### Surgery

All patients underwent typical thyroid dissection. Total or near-total thyroidectomy was performed for bilateral PTC in 155 patients. Unilateral thyroidectomy plus isthmectomy were performed for unilateral PTC in 175 patients, and subtotal thyroidectomy was added if benign nodules were found in the contralateral lobe. CND was performed routinely on the affected side. CND on the contralateral side was performed when any of the central lymph nodes were found to be suspicious on preoperative imaging exmaination or upon intraoperative inspection or palpation. LND was performed only if preoperative fine needle aspiration cytology or intraoperative frozen section biopsy revealed evidence of metastasis.

### Cervical lymph node removal

Soft tissue above the thyroid isthmus and anterior to the cricothyroid membrane between the cricothyroid muscles was removed and labeled as the DLN. The remaining central lymph nodes were subdivided into pretracheal, ipsilateral and contralateral paratracheal groups according to their locations. LND was performed to remove the lateral level II, III, IV, and V nodes.

### *BRAF*^V600E^ mutation detection

Tumor samples were excised, immediately put into sterile vials containing chilled phosphate-buffered saline (pH = 7.2), sent to the biomolecular laboratory, and frozen at −80°C for molecular investigations.

DNA was extracted with a DNA extraction kit (Promega Corporation, USA), and BRAF gene exon 15 was detected with a BRAF mutant gene detection kit (Amoy Diagnostics Co., LTD, China) and an ABI7500 real-time polymerase chain reaction amplifier (Applied Biosystems, USA). For the amplification of exon 15 of BRAF, the forward primer 5′-TCATAATGCTTGCTCTGA TAGGA-3′ and reverse primer 5′-GGCCAAAAATTT AATCAGTGGA-3′ were used. All BRAF^V600E^ mutation detection procedures and results analysis were conducted by technicians in our institution’s biomolecular laboratory.

### Statistical analysis

SPSS v. 18.0 (SPSS, Inc., Chicago, IL, USA) was used for all statistical analyses. Categorical and continuous data were tested with the Student *t*-test, chi-square test, or Fisher exact test. Variables found to be significantly different between groups in the univariate analysis were included in the multivariate logistic regression analysis. Differences were considered significant when *P* < 0.05.

## Abbreviations

DLN: Delphian lymph node
PTC: papillary thyroid cancer
CNNM: central neck node metastases
CNNs: Central neck nodes
CND: central neck dissection
LND: lateral neck dissection
ETE: extrathyroid extension
LNNM: lateral neck node metastases.
